# Use of GnRH for Synchronization of the Follicular Wave in Assisted Reproductive Technologies in Sheep: A Preliminary Study

**DOI:** 10.3390/ani10071208

**Published:** 2020-07-16

**Authors:** Aina Año-Perello, Zurisaday Santos-Jimenez, Teresa Encinas, Paula Martinez-Ros, Antonio Gonzalez-Bulnes

**Affiliations:** 1 Departamento de Produccion y Sanidad Animal, Facultad de Veterinaria, Universidad Cardenal Herrera-CEU, CEU Universities, C/ Tirant lo Blanc, 7. 46115 Alfara del Patriarca Valencia, Spain; ainaanyo64@gmail.com (A.A.-P.); bulnes@inia.es (A.G.-B.); 2 Departamento Ciencias Medico Veterinarias, Universidad Autónoma Agraria Antonio Narro, Torreón, 25315 Coahuila, Mexico; mvz_zusan@hotmail.com; 3 Departamento de Farmacologia y Toxicologia Facultad de Veterinaria, UCM, Ciudad Universitaria s/n, 28040 Madrid, Spain; tencinas@ucm.es; 4 Departamento de Reproduccion Animal, Instituto Nacional de Investigación y Tecnología Agraria y Alimentaria (INIA), Avda. Puerta de Hierro s/n. 28040-Madrid, Spain

**Keywords:** assisted reproduction, estrus synchronization, follicle, GnRH, sheep

## Abstract

**Simple Summary:**

The present study aimed to set up a short-term protocol for synchronization of follicular wave emergence in sheep, concomitant with estrus synchronization, which would improve ovarian response in assisted reproductive technologies. Administration of a single dose of gonadotrophin-releasing hormone (GnRH), concomitant with the insertion of a progesterone-loaded controlled internal drug release (CIDR) device, caused regression of gonadotrophin-dependent follicles in all test sheep, with 70% of them initiating a new follicular wave and the remaining showing non-dominant follicles.

**Abstract:**

The present study aimed to set up a short-term protocol for synchronization of follicular wave emergence in sheep, concomitant with estrus synchronization, which would improve ovarian response in assisted reproductive technologies. Administration of a single GnRH dose, concomitant with the insertion of a progesterone-loaded CIDR device, caused regression of gonadotrophin-dependent follicles ≥4 mm in all the GnRH-treated sheep and in around 80% of the controls treated only with CIDR (*p* < 0.05). Similar percentages of ewes lost all follicles (around 70%) or only the largest one (around 30%) in both groups. Hence, 54.1% and 70% of the sheep lost all large follicles and initiated a new follicular wave in the control and GnRH groups, respectively (*p* < 0.05). The remaining sheep showed follicles that were still not dependent of luteinizing hormone (LH). So, in fact, all the sheep had non-dominant follicles after treatment. In conclusion, a treatment including GnRH at CIDR insertion would offer a time- and cost-efficient protocol for inducing follicular turnover and synchronizing a new follicular wave at any stage of the estrous cycle.

## 1. Introduction

In sheep, as in other ruminant species, the use of assisted reproductive technologies like artificial insemination (AI) and multiple ovulation and embryo transfer (MOET) constitute the most effective and efficient strategies to improve the number of offspring from valuable animals and, therefore, to increase genetic gain. The success of both reproductive technologies is largely dependent on the interaction between the ovarian status and the hormonal treatments needed for allowing their application (progesterone for inducing and synchronizing estrus occurrence and gonadotrophins for inducing follicular growth and ovulation) [1,2,3].

Traditionally, estrus synchronization in sheep is based on the administration of intravaginal progestative treatments for 12 to 14 days [4]. These protocols were developed during the 1950s and 1960s from a “corpus-luteum-focused” perspective. The objective was to use exogenous progesterone to imitate the role of the corpus luteum in the control of the estrous cycle, avoiding the occurrence of ovulations during progesterone supplementation and allowing them at progesterone removal. However, such protocols neglected to take into account the follicle—destined to release an oocyte able to result in a pregnancy and to develop in a corpus luteum supporting such a pregnancy. In fact, 12 to 14 days of treatment may affect the quality of preovulatory follicles [5] and, therefore, the fertility of oocytes and the function of corpora lutea [6,7]. The basis for the administration of progesterone is the decrease in secretion of luteinizing hormone (LH) that prevents the occurrence of estrus, the preovulatory LH surge, and ovulation until the progestative treatment is withdrawn [8]. The decrease in LH secretion induced by exogenous progesterone causes large follicles (gonadotrophin-dependent follicles, ≥4 mm in size in the case of sheep [3,9]) present in the ovaries to become atretic, allowing follicular turnover and the emergence of a new follicular wave [10,11]. However, sometimes, progesterone release after 12–14 days of treatment is too low to adequately suppress LH. The failure in suppressing LH secretion causes abnormal follicular development with large, persistent follicles [5,12]. Afterward, the ovulation of these aged follicles affects fertility [13,14].

Currently, such knowledge is giving way to “follicle-focused” protocols [15]. These protocols consist of short-term progestative treatments (5–7 days), which are equally as effective as long-term protocols for inducing preovulatory events and ovulation [16,17], with even a higher fertility [2,18]. Short-term protocols aim to synchronize estrus and ovulation but also the follicular wave from which the ovulatory follicles arise. The insertion of progesterone treatment abruptly decreases LH secretion and induces atresia of large follicles and the emergence of a new wave, giving way to preovulatory follicles 5 to 6 days later [19]. Such an effect, as previously described, is lost in long-term protocols in which, moreover, the decrease in progesterone release during the time of treatment affects the quality of preovulatory follicles [5].

Synchronization of the follicular wave is interesting for AI but even more attractive for MOET protocols, in which the yields are largely dependent on the ovarian follicle population of the donor female at the beginning of the superovulatory follicle-stimulating hormone (FSH) treatment. The presence of dominant follicles diminishes the ovarian response to the treatment, but it mainly diminishes the number of transferable embryos [3]. Hence, treating animals in the absence of dominant follicles is crucial. However, 70 to 85% of donors typically have a dominant follicle at the time of the first FSH administration [20,21]. Thus, synchronizing the emergence of a new follicular wave in donors with the start of the superovulatory treatment, when there are no large follicles yet, constitutes a successful procedure. One option is the “Day 0 Protocol”, which consists of applying the superovulatory treatment during the first follicular wave of the cycle, the wave emerging on day 0 after ovulation, by synchronizing to the previous cycle [22].

Our hypothesis is that the efficiency of synchronizing the follicular wave and ovulatory events, both for AI and MOET, may be improved by gonadotrophin-releasing hormone (GnRH) administration at the beginning of the progesterone treatment (and the FSH treatment in MOET protocols). The administration of a single dose of GnRH at progesterone insertion would induce regression of any large follicle and, therefore, the emergence of the subsequent new follicular wave, without needing a prior treatment for synchronizing the first ovulation as in the “Day 0 Protocol”. There is no direct evidence supporting our hypothesis, so we will assess the effects of insertion of progesterone-loaded controlled internal drug release (CIDR) devices, with or without a concomitant GnRH injection, on the fate of gonadotrophin-dependent follicles.

## 2. Materials and Methods

### 2.1. Animals and Ethical Issues

The experiment involved 64 non-lactating Segureña meat ewes, 2 to 5 years old, from the experimental flock of the CEU Cardenal Herrera University in Naquera (Valencia, Spain; latitude 39° N). The experiment was performed during the reproductive season (February) according to the Spanish Policy for Animal Protection RD53/2013, which meets the European Union Directive 2010/63/UE about the protection of animals used for research and was specifically assessed and approved by the CEU Cardenal Herrera Committee of Ethics in Animal Research (Report CEEA17/019).

### 2.2. Experimental Design

On day 0 of the experimental period, all the ewes were treated with one intravaginal controlled internal drug release device (0.35 g of progesterone; CIDR^®^ Ovis, Zoetis, Madrid, Spain) and divided into two groups which, at CIDR insertion, received either a single dose of 50 µg of GnRH (Acegon^®^, Lab. Syva, Leon, Spain; group GnRH, n = 24) or saline solution (group SS, n = 40).

Presence, number, and size of all ≥ 4 mm follicles were determined by transrectal ultrasonography (Aloka SSD500 fitted to a 7.5 MHz linear-array probe, Aloka Co., Ltd., Tokyo, Japan), just prior to CIDR insertion on day 0. Concurrently, the presence of corpora lutea and, therefore, ovulatory cyclic activity of the animals was assessed in the same screening. The ultrasonographic screening (assessing again the presence, number, and size of all ≥ 4 mm follicles) was repeated on the following day (day 1) and detected follicles in regressing phase (which decreased in diameter or disappeared) and follicles that remained stable or grew.

Concomitant with ovarian ultrasonography at CIDR insertion and 24 h later, samples of 5 mL of jugular blood were collected with heparinized vacuum blood evacuation tubes (Vacutainer^®^ Systems Europe, Becton Dickinson, Meylan Cedex, France) for assessment of plasma estradiol concentrations. Blood samples were immediately centrifuged at 2000× *g* for 15 min. Thereafter, the plasma was stored at −20 °C until assayed for estradiol-17β determination. Such determination was performed, after sample extraction, by using an enzyme immunoassay kit (Demeditec Diagnostics GmbH, Kiel-Wellsee, Germany). Sensitivity was 1.4 pg/mL, and intra-assay variation coefficient was 5.7%.

### 2.3. Statistical Analysis

The effects of treatment on follicle dynamics and plasma estradiol concentration were assessed by analyses of variance (ANOVA) and chi-square tests, after normality testing of the data, by using SPSS^®^ 22.0 (IBM Corporation, New York, NY, USA). All results were expressed as mean ± S.E.M., and the statistical significance was accepted from *p* < 0.05.

## 3. Results

The first ultrasonographic observation at CIDR insertion (day 0) showed that around 90% of the sheep showed the presence of follicles ≥ 4 mm in size, such a proportion was equally high and comparable in both groups (Table 1). The ultrasonographic observation performed on the following day (day 1) showed that CIDR insertion caused the regression of large follicles in 81.1% of the sheep in the control group, with all of them showing a significant decrease in plasma estradiol concentrations, from mean values of 4.6 ± 0.7 pg/mL to mean values of 1.8 ± 0.6 pg/mL (*p* < 0.05).

In contrast, the administration of a GnRH dose concomitant with CIDR insertion induced follicle regression in all the treated sheep (*p* < 0.05). This was confirmed by a significant decrease in estradiol concentrations from 4.2 ± 0.7 pg/mL to mean values of 1.6 ± 0.6 pg/mL (*p* < 0.05).

The percentage of sheep losing all follicles or only the largest one was similar in both groups. Hence, 54.1% and 70% of the sheep in the control and GnRH groups, respectively, showed regression on day 1 of the large follicles found on day 0 (*p* < 0.05), but none of the others showed follicles larger than 4 mm on day 1.

## 4. Discussion

Our results indicate that the insertion of a progesterone-loaded CIDR device caused the regression of large follicles in around 80% of the treated sheep. The regression of all gonadotrophin-dependent follicles was complete in around two-thirds of these ewes, while some such follicles remained in the other sheep. In consequence, around 54% of the ewes initiated a new follicular wave. These results support earlier studies indicating that exposure to high levels of exogenous progesterone or progestogens affects the viability of large follicles and increases follicular turnover [3,9,23]. In the current study, elimination of large follicles was improved by the addition of a single GnRH dose at CIDR insertion, which induced the onset of a new follicular wave in around 70% of the sheep. The remaining sheep showed follicles that were gonadotrophin-responsive but still non-LH-dependent. So, in fact, all the sheep had non-dominant follicles after CIDR and GnRH treatments.

The removal of large follicles and the emergence of a new follicular wave in these ewes imply the growth of the preovulatory follicles in this wave during the period of permanence of the short-term progesterone treatment (five days) to reach the ovulatory size at CIDR withdrawal [18]. In fact, previous studies indicate that ovulatory follicles emerge from the pool of small gonadotrophin-responsive follicles at the start of their follicular wave [24,25] and actively grow for the following four days [26,27] to reach their maximum diameter around the fifth day [28]. Moreover, the use of CIDR assures high serum plasma progesterone concentrations during the development of the ovulatory follicle, which is highly beneficial for adequate oocyte health at ovulation [29,30]. In summary, such a scenario would assure the best condition for optimal fertility after AI.

The proposed treatment, including GnRH at CIDR insertion, is also highly useful for sheep MOET programs. The use of GnRH, inducing the regression of large follicles and the emergence of new preovulatory follicles, implies that most of the preovulatory follicles growing after its injection emerge from the pool of gonadotrophin-responsive follicles. Previous studies have found that the absence of large follicles and a high number of gonadotrophin-responsive follicles are the optimal follicular population when starting a superovulatory FSH protocol in sheep [20]. In fact, our proposed protocol would set up a situation similar to day 0 of the estrous cycle. This is considered, as previously described, the best scenario for applying assisted reproductive techniques and led to the so-called “Day 0 Protocol”, which significantly improves embryo yields by using the first wave of the estrous cycle [22]. Our protocol would set up the same scenario with only a single injection.

## 5. Conclusions

Protocols including a single dose of GnRH at CIDR insertion can offer a time- and cost-efficient protocol for optimizing follicular status in AI and MOET protocols. GnRH can increase the response of ewes, improving the follicular turnover when compared with ewes treated with CIDR alone, without the need of a previous synchronization of the cycle and the ovulation of the dominant follicle that is needed in the “Day 0 Protocol”.

## Figures and Tables

**Table 1:** Percentage of sheep showing presence of ≥ 4 mm follicles at CIDR insertion (day 0) and effects of CIDR insertion alone (control group) or combined with a single GnRH dose (GnRH group) on regression of such follicles on day 1.

Percentage of Sheep	Control	GnRH
Sheep with large follicles at CIDR insertion (Day 0) Mean number of large follicles in them	92.5% (37 of 40) 1.7 ± 0.1	83.3% (20 of 24) 1.9 ± 0.2
Sheep with regression of large follicles on Day 1	81.1%^a^ (30 of 37)	100%^b^ (20 of 20)
Sheep with regression of all large follicles on Day 1	66.7% (20 of 30)	70% (14 of 20)
Sheep with only the largest follicle regressing on Day 1	33.3% (10 of 30)	30% (6 of 20)

Different superscripts indicate significant differences among treatments (a ≠ b: *p* < 0.05).
